# *Scabiosa* Genus: A Rich Source of Bioactive Metabolites

**DOI:** 10.3390/medicines5040110

**Published:** 2018-10-09

**Authors:** Diana C. G. A. Pinto, Naima Rahmouni, Noureddine Beghidja, Artur M. S. Silva

**Affiliations:** 1 Department of Chemistry and QOPNA, University of Aveiro, Campus de Santiago, 3810193 Aveiro, Portugal; rahmouni_na@yahoo.fr (N.R.); artur.silva@ua.pt (A.M.S.S.); 2 Unité de Recherche et Valorisation des Ressources Naturelles, Molécules Bioactives et Analyse Physico-Chimiques et Biologiques, Université des Frères Mentouri Constantine 1, Constantine, Algérie; nourbeghidja@yahoo.fr

**Keywords:** *Scabiosa*, flavonoids, iridoids, pentacyclic triterpenoids, antioxidant, anti-inflammatory, antibacterial, anticancer

## Abstract

The genus *Scabiosa* (family Caprifoliaceae) is considered large (618 scientific plant names of species) although only 62 have accepted Latin binominal names. The majority of the *Scabiosa* species are widely distributed in the Mediterranean region and some *Scabiosa* species are used in traditional medicine systems. For instance, *Scabiosa columbaria* L. is used traditionally against diphtheria while *S. comosa* Fisch. Ex Roem. and Schult. is used in Mongolian and Tibetan traditional medical settings to treat liver diseases. The richness of *Scabiosa* species in secondary metabolites such as iridoids, flavonoids and pentacyclic triterpenoids may contribute to its use in folk medicine. Details on the most recent and relevant pharmacological in vivo studies on the bioactive secondary metabolites isolated from *Scabiosa* species will be summarized and thoroughly discussed.

## 1. Introduction

From the pharmacological perspective, plants are a treasure. In fact, the plant itself or its secondary metabolites are the source of useful drugs. They still are the main source of bioactive compounds that can be used directly in remedies, or can inspire the synthesis of more active derivatives [1]. Accordingly, the scientific community has renewed its interest in pharmacologically active natural compounds trying to find cures for many diseases. Moreover, herbal remedies are also enjoying a revival in developed countries, and in many countries, traditional medicine is the first option, or the only one, for health maintenance and disease prevention or treatment. In this context, *Scabiosa* species are significant due their applications in traditional medicine systems but also due to their richness in bioactive compounds.

Some authors indicate that there are 100 species of *Scabiosa* [2]. However, from the 618 scientific plant names listed, only 62 are accepted species names, with the others being synonyms and/or unresolved names [3]. Currently, genus *Scabiosa* belongs to the family of Caprifoliaceae, although in previous reports appears included in the Dipsacaceae family. However, due to morphological and molecular phylogenetic analyses, Dipsacaceae is no longer recognised as a family and their species are currently placed in the family Caprifoliaceae [4]. These changes in the species taxonomy, although understandable, may lead to several confusions in the literature and consequently increase difficulties to the phytochemical researchers (usually chemists).

All the botanic names referred herein were confirmed in “The Plant List” database [3] and the full-accepted binominal Latin scientific name will be displayed in the first citation while in subsequent citations *Scabiosa* will be indicated by the first capital letter and the authors’ names will be omitted.

The genus *Scabiosa* L. is considered a large taxonomically complex genus with several species distributed in the Mediterranean Basin, Asia and southern Africa [5,6]. *Scabiosa* species are annual plants with basal leaf rosettes and leafy stems. They are mostly shrubs with variation in size from 10 cm, such as in *Scabiosa stellata* L. case [7], to 60 cm, in the case of *Scabiosa atropurpurea* L. [8]. Their flowers have crowded small heads with colours ranging from white to purple, which is why some are used as ornamental plants. *Scabiosa* species are also used as medicinal plants, and phytochemical studies revealed that they are able to produce interesting secondary metabolites some of which have proved to be promising therapeutic agents. Thus, herein we report and discuss the information on traditional medicine applications, bioactive natural compounds isolated from *Scabiosa* species, highlighting the more relevant metabolites and/or bioactivities.

## 2. *Scabiosa* Genus: Traditional and Pharmacological Applications

There are a few reports indicating that species from the genus *Scabiosa* are used in traditional medicine. However, it should be highlighted that several species need some taxonomic confirmations. For example, *Scabiosa succisa* L., which is reported to be used in the treatment of bronchitis, influenza and asthma [9], is also considered a synonym of *Succisa pratensis* Moench., the current accepted name for the species [10]. Besides, several publications still use the former family name Dipsacaceae. Despite the mentioned drawbacks, the use of *Scabiosa* species in traditional medicine systems is happening, particularly in China [11]. For instance, *Scabiosa atropurpurea* L. is used in Catalonia to treat measles and furuncles [12] and it is also a recognized medicinal plant in France [13]. Another species with several references is *Scabiosa columbaria* L. which is used to treat diphtheria [14] and respiratory infections, high blood pressure and uterine disorders [15,16], among others. Three other species are also reported to have medicinal uses, *Scabiosa stellata* L. is used to treat heel cracks [17] and both *Scabiosa tschilliensis* Grüning and *Scabiosa comosa* Fisch. Ex Roem. and Schult. are used to treat liver diseases [11]. Recently, the natural medicine Gurigumu-7, used in traditional Mongolian medicine and including in its composition the flowers of *S. comosa*, was evaluated for its hepatoprotective effect. Moreover, not only the beneficial effect and consequently clinical efficacy was proved but also that the more active fraction is the methanolic one, suggesting that the active compounds are the polar ones [18].

Studies to confirm the medicinal use and/or to find the pharmacological properties of *Scabiosa* species are reduced and mainly concerned with extracts activities. Moreover, the studied species are also restricted and toxicological evaluations were not accomplished. An overview of the evaluations carried out revealed that the majority are in vitro assessments of the antimicrobial and the antioxidant activities. Some in vitro cytotoxic evaluations were also reported, as well as the less common activities, such as anti-HCV [19], anti-tyrosinase [2] and acetylcholinesterase inhibition [20].

Although the biological assessments are scarce, some can be mentioned; for example, the ethanolic extract of *S. atropurpurea*, plant is used in Peru as an antibacterial remedy and its capacity to inhibit *Staphylococcus aureus* was evaluated. The minimum inhibitory concentration (MIC) obtained (32 mg/mL) indicates that the extract activity is not strong (only values below 5 mg/mL are considered strong) but it is an indication that it might have active metabolites [21]. As far as we are aware, the only in vivo study was performed with *S. atropurpurea* ethanolic extract, which demonstrated antihyperglycaemic, hepatoprotective and antioxidant activities [22].

*Scabiosa hymettia* Boiss. and Spruner: although not a medicinal plant it was evaluated to establish its antimicrobial value. The methanolic and chloroform extracts were evaluated against Gram-(+) and Gram-(−) bacteria and human pathogenic fungi. Both extracts showed moderate activity against the microorganisms used [23]. *Scabiosa columbaria* was also investigated for its antimicrobial activity and, therefore, this validated its use in traditional medicine [24]. Other medicinal plants such as *S. comosa* and *S. tschilliensis* were demonstrated to have in their chemical composition metabolites with antioxidant and anti-HCV activities. These results also validate their traditional use in several medicine systems [19]. Furthermore, the antioxidant capacity of *S. tschilliensis* was recently proved by other authors [25]. In the beginning of this year, another medicinal plant, *S. stellata*, was investigated in order to find its antioxidant, antibacterial and anti-tyrosinase power. Although the extracts exhibit some activity, it is clear that the pure compounds are more active [2]. Our final examples are the cases of *Scabiosa prolifera* L., for which in vitro antioxidant and cytotoxic activities were demonstrated [26], and *Scabiosa arenaria* Forssk., for which acetylcholinesterase inhibition, antioxidant activity [20] and antimicrobial activity [27] were reported. The problem with these results is in the species identification, both *S. prolifera* and *S. arenaria* are unresolved names [3].

## 3. Structural Pattern of the Secondary Metabolites Isolated from *Scabiosa* Species

**Figure 1:**
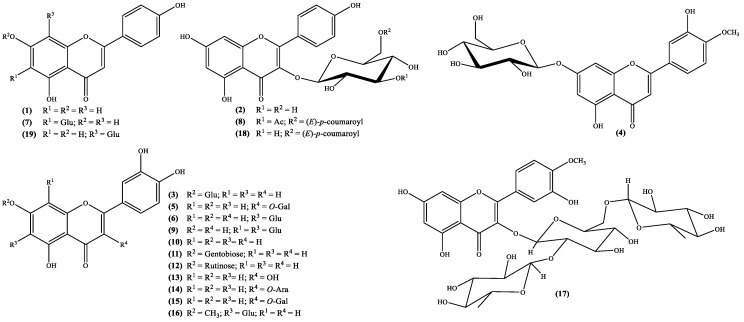
Flavonoids isolated from the genus *Scabiosa* (Ara = arabinose; Gal = galactose; Glu = glucose).

**Figure 2:**
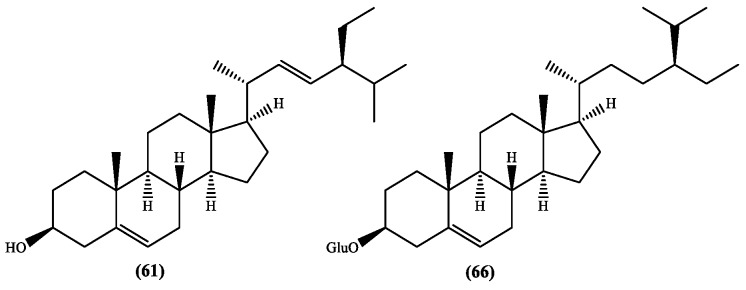
Steroids isolated from the genus *Scabiosa* (Glu = glucose).

**Figure 3:**
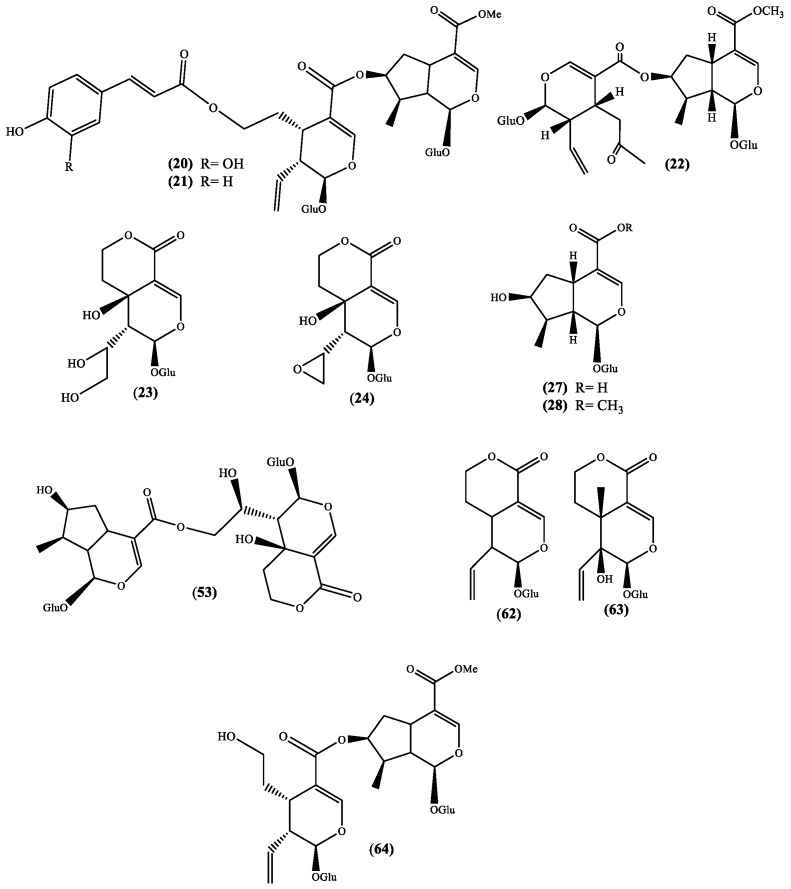
Iridoids isolated from the genus *Scabiosa* (Glu = glucose).

**Figure 4:**
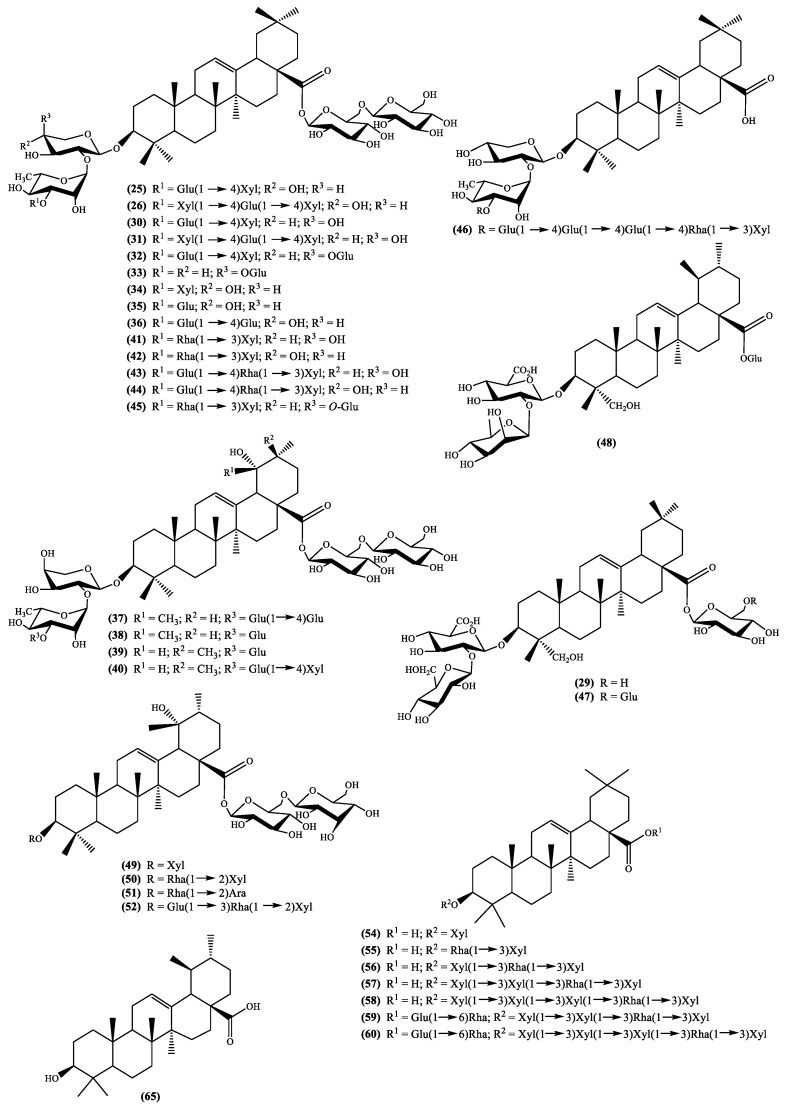
Pentacyclic triterpenoids isolated from the genus *Scabiosa* (Ara = arabinose; Gal = galactose; Glu = glucose; Rha = rhamnose; Xyl = xylose).

To understand the pharmacological activity of the genus *Scabiosa* it is essential to perform detailed and extensive phytochemical investigations. In fact, the isolation of secondary metabolites and evaluation of their biological activities including the study of their mechanisms of action are important to validate (or not) the traditional medicine based in this species and, ultimately, to find new drugs. Up to date, only a few *Scabiosa* species were subjected to phytochemical studies, however, a wide spectrum of secondary metabolites has been identified and allowed to confirm that this genus species is rich in flavonoids and terpenoids. Herein, profiling analysis, although valuable research works, will not be discussed; this manuscript will be focused in the isolated secondary metabolites, emphasizing the flavonoid, iridoid and triterpenoid derivatives. The names of these constituents and the plants from which they were isolated are listed in Table 1 and their structures are depicted in Figure 1, Figure 2, Figure 3 and Figure 4.

It should be also pointed out that only the phytochemical studies involving accepted *Scabiosa* species will be presented. In fact, this option may cause the elimination of some phytochemical studies but it is also a fact that ambiguous identifications automatically invalidate the reported results.

Important biological properties, such as anticancer [38], anti-inflammatory [39] and antioxidant [40] activities, just to mention a few [41] are the reason why flavone derivatives are included amongst the most important secondary metabolites. Subsequently the occurrence of these metabolites both as aglycones and glycosides in *Scabiosa* genus (Figure 1; Table 1) can explain and/or confirm the claimed medicinal properties. The structures analysis (Figure 1) demonstrates that the flavone derivatives isolated from species of the genus *Scabiosa* are mostly derivatives of apigenin, diosmetin and luteolin, which are polyhydroxylated flavones. The other derivatives reported are flavonol types such as kaempferol and quercetin derivatives, also polyhydroxylated compounds.

The occurrence of flavonoids in the *Scabiosa* genus is also important from the taxonomical point of view as has been shown by Perdetzoglou et al. [28], where the flavonoid types of compounds were used to establish that *Scabiosa argentea* L. and *Scabiosa tenuis* Spruner ex Boiss. are taxonomically independent species [28].

In other cases, such as the species *S. hymettia* were isolated two interesting kaempferol derivatives, astragallin (kaempferol 3-*O*-β-D-glucoside) **2** and the new natural compound kaempferol-3-*O*-[3-*O*-acetyl-6-*O*-(*E*)-*p*-coumaroyl]-β-D-glucoside **8** (Figure 1; Table 1), which may explain the plant antimicrobial activity [23]. Most recently several flavonoids were isolated from *S. stellata* [2,29], not only are found for the first time in the genus, but also confirm its richness in these metabolites. Interesting derivatives, such as compounds **5**, **9**, **16**, **17** and **18** (Figure 1; Table 1) may be responsible for the plant antioxidant activity [2,29]. Biological activities found in *S. atropurpurea* [22] could also be related to its flavonoid content, mostly luteolin derivatives, from which luteolin-7-*O*-rutinoside **12** (Figure 1; Table 1) can be highlighted because it was found for the first time in the genus [22].

Conversely, the recent work of Al-Qudah et al. [26], where the species identification is not properly presented, cannot be highlighted here, although the authors claimed the isolation of flavonoids that might explain the plant antioxidant activity.

As far as we are aware only stigmasterol **61** and β-sitosterol-β-d-glucoside **66** (Figure 2) were isolated from *S. stellata* [37]. Lipophilic profiles could show the presence of steroid derivatives, but those works are not included in this review because herein are just referred the isolated and fully characterized metabolites. Nevertheless, the presence of β-sitosterol derivatives seems to be important due to their recognised biological properties and potential use in treatment of various illnesses [42], but also stigmasterol seems to be a potential therapeutic agent for neurodegenerative diseases [43]. Therefore, *S. stellata* can be a source of these important secondary metabolites.

Several biological activities are also attributed to iridoids [44,45] and this fact improves the value of *Scabiosa* species, which are recognized to produce several iridoid derivatives (Figure 3 and Table 1). The works that reported these metabolites are recent and the plants are well identified allowing their recommendation for further studies, in particular the species *S. hymettia* [23] and *Scabiosa variifolia* Boiss. [33], which are not reported as medicinal plants, but certainly can be a source of important bioactive compounds. In the cases of *S. atropurpurea* [32] and *S. stellata* [2] we are in the presence of medicinal plants, thus these studies are always recommended to validate their medicinal use. The recent reported new natural sylvestroside I 64 and derivatives, 7-*O*-(*E*-caffeoyl)sylvestroside I **20** and 7-*O*-(*E*-*p*-coumaroyl)sylvestroside I **21** (Figure 3) [2] can be highlighted, not only because they are new compounds but also due to the presence of a cinnamic acid moiety. This moiety is an important fragment of chlorogenic acids, which are known natural compounds and recognized for their important biological activities [46]. In fact, the chlorogenic derivatives 3,5-*O*-dicaffeoylquinic acid and 4,5-*O*-dicaffeoylquinic acid were recently isolated from *S. stellata* [2,29] and, to find reports about the isolation of these metabolites we have to go back to the work of Zemtsova et al. where they claimed the isolation of chlorogenic acid from *Scabiosa olgae* Albov [30] and from *Scabiosa bipinnata* C. Koch [47]. Another relevance of the sylvestroside I **64** and derivatives isolated is the moderate cytotoxic activity (IC_50_ 35.9 µg/mL) against brosarcoma cell lines (HT1080) shown by 7-*O*-(*E*-caffeoyl) sylvestroside I **20** [2], result that once again point out the *S. stellata* value as source of interesting secondary metabolites.

Although the number of *Sacabiosa* species studied from the phytochemical point of view is scarce, one thing is clear; this genus species produces terpenoids such as the above mentioned but also pentacyclic triterpenoids. Terpenoids is one of the largest and most diverse classes of secondary metabolites produced by plants where they play several functions [48], but they are also used by humans in the pharmaceutical industry [49]. From the biological perspective, pentacyclic triterpenoids can be highlighted due to their anti-inflammatory [50] and the antitumor [51,52] activities, but their natural occurrence is also extensive [53].

The richness of the *Scabiosa* species in pentacyclic triterpenoids seems to be obvious (Figure 4 and Table 1) and it is evident that almost all the isolated pentacyclic triterpenoids are saponins. This seems to be a characteristic of the genus *Scabiosa*, being the main aglycones oleanolic and pomolic acids, with glucose, xylose, rhamnose and arabinose as sugars (Figure 4). It should be stressed that, among the several biological activities reported for oleanolic acid [54], its potential as a cancer therapy drug [55] is the most significant. Pomolic acid, is a less studied pentacyclic triterpenoid, but nevertheless showed anti-HIV activity [54].

The literature survey demonstrates that *S. tschilliensis* can be a good source of pentacyclic triterpenoids acids, such as oleanolic and pomolic (Figure 4 and Table 1), through a cleavage of the sugar moieties. Moreover, the presence of these secondary metabolites may explain the plant medicinal use.

*Scabiosa rotata* M.Bieb., as far as we are aware, is not used in folk medicine but is also a good source of pomolic acid (Figure 4 and Table 1). On the other hand, *S. songarica* Schrenk and the medicinal plant *S. stellata* can be regarded as good sources of oleanolic acid (Figure 4 and Table 1).

To the extent that we could investigate, *S. stellata* seems to be the species presenting more diversity in the saponins aglycones. Along with oleanolic acid, ursolic acid and hederagenin derivatives were isolated (Figure 4 and Table 1).

## 4. In Vivo Assessments of Nominated Metabolites

The aim of this review is an update on the information about *Scabiosa* species secondary metabolites as well as their biological potential. In fact, from the above-mentioned secondary metabolites, some (e.g., iridoids, flavonoids and pentacyclic triterpenoids) can be highlighted, due to their recognized activities. Unfortunately, many studies involve extracts or are in vitro assessments. Herein, we select the most interesting secondary metabolites or their aglycones for which in vivo assessments were reported. Consequently, the activities mentioned herein will be also limited to the ones that were evaluated in vivo.

### 4.1. Flavonoid-Type Metabolites

The analysis of the flavonoids isolated from *Scabiosa* genus (Figure 1 and Table 1) point toward that their occurrence is in the glycoside form. However, the main aglycones (apigenin, diosmetin, kaempferol, luteolin and quercetin) biological potential is well known. Tamarixetin, the aglycone of compound **17**, may be the less known one and consequently less studied. Nevertheless, its in vitro ability to inhibit the proliferation of leukemia cells [56] and enhancement of the Ca^2+^ transients, both in vitro and in vivo [57], have been demonstrated. Moreover, the 3-*O*-β-d-glucopyranoside derivative reveals ability to, in vivo, inhibit the matrix metalloproteinase-9, that can be regarded as potential drug to treat gastric ulceration [58].

Tiliroside **18** (Figure 1) is a kaempferol glycoside derivative whose structure was elucidated in 1964 [59] and was found first in *Tilia* species but nowadays is present in several plants. Through the years, this flavonol type compound gathered the scientific community’s interest and interesting in vitro activities were reported. These include antidiabetic activity [60,61], inhibition of neuroinflammation in murine cultured microglial cells BV2 (cells immortalized after infection with a recombinant retrovirus) [62] and antiproliferative properties on human breast cancer cell lines (T47D and MCF7) [63]. The in vivo studies are less, nonetheless some can be emphasized. For example, Barbosa et al. [64] in their efforts to validate the use of medicinal plants to treat diarrhea, performed some in vivo antiprotozoal assessments, against the protozoa *Giardia lamblia*. Among the tested flavonoids is tiliroside **18**, for which an ED_50_ value of 1.429 μmol/kg was obtained, a value that is similar to the one obtained with metronidazole (ED_50_ 1.134 μmol/kg), one of the positive controls used in the study. Nevertheless, is less active than the other positive control, emetine (ED_50_ 0.351 μmol/kg) [64]. The tiliroside **18** anti-inflammatory potential was also evaluated and an in vivo study showed that it can inhibit the mouse paw oedema (ED_50_ = 35.6 mg/kg) and the mouse ear inflammation (ED_50_ = 357 Ag/ear) [65]. The inhibition of the enzymatic and non-enzymatic lipid peroxidation (IC_50_ = 12.6 and 28 μM, respectively) and the scavenger properties, both in the superoxide radical (IC_50_ = 21.3 μM) and in the DPPH assay (IC_50_ = 6 μM), suggest that tiliroside **18** anti-inflammatory activity is related to its antioxidant activity [65]. More recently, Jin et al. [66] proposed that tiliroside **18** anti-inflammatory activity can be explained through its involvement in the downregulation of the inducible nitric oxide synthase (iNOS) and cyclooxygenase-2 (COX-2) protein expression levels and in the inactivation of mitogen-activated protein kinase (MAPK) signaling pathway [66].

Finally, the in vivo antihypertensive and vasorelaxant effects of tiliroside **18** were also evaluated and the mechanism of action studied [67]. The findings suggest that tiliroside **18** induces a decrease in blood pressure and through the blockage blockade of Ca^2+^ channels (Ca_V_ 1.2) in vascular smooth muscle cells (VSMCs) promotes the vasorelaxant effect [67].

As far as we could find, vitexin **19** (Figure 1) was the first *C*-glycoside flavonoid isolated from natural sources [68], and accordingly to the publication, was isolated from *Vitex littoralis*, which is a synonym of *Vitex parviflora* A.Juss. [3]. Vitexin **19**, an apiginin glycoside, is among the flavonoid derivatives found in *Scabiosa* species, the most studied one. It is included in structure activity relationships [69] or even used as inspiration to develop new active compounds [70].

Although, herein we are disclosing the more recent in vivo studies, it is a surprise that this metabolite’s in vivo evaluation started in 1995 with a study of its antithyroid effects, concluding that can be used to prevent goiter [71]. The more recent studies include antimicrobial activity against *Pseudomonas aeruginosa*, for which the vitexin **19** activity was moderated [72], cardioprotective effects, which demonstrated that vitexin **19** mitigated myocardial ischemia reperfusion injury and suppressed apoptosis and autophagy in myocardium cells [73], and its protection of dopaminergic neurons, which suggests its use in Parkinson’s disease therapy [74].

As expected, anti-inflammatory in vivo studies were also recently reported; from those we emphasize the Rosa et al. work [75] due to the detailed analysis that included the cytotoxicity evaluation. The authors tested several doses and confirm that vitexin **19** was not cytotoxic towards macrophage normal cell line (RAW 264.7) and established that its anti-inflammatory action was due to the inactivation of pro-inflammatory pathways [75]. In fact, the anti-inflammatory activity of vitexin **19** seems to be the related with its possible use to alleviate epilepsy [76].

As a final point, the anticancer evaluations suggest that vitexin **19** antitumor efficacy can be related to its ability to activate the c-Jun NH_2_-terminal kinase-signaling pathway. Consequently, vitexin **19** can be regarded as a possible drug to treat hepatocellular carcinoma [77] or colorectal cancer [78,79]. Moreover, a recent detailed review [80] disclosed the potential of this flavonoid towards is use in cancer therapy.

Taken together, the above-mentioned findings seem to clearly state that *Scabiosa* species produce important bioactive flavonoids that can explain their medicinal use but also can incentive more investigations.

### 4.2. Iridoid Type Metabolites

Likewise, the flavonoids, the iridoids isolated from *Scabiosa* genus (Figure 3 and Table 1) are glycosylated. Actually, a recent study showed that these glycosides can be considered responsible for the hepatoprotective effect of the Gentianaceae herbs extracts [81], extracts that are commonly used as food additives. An in vitro assay established that a fraction of the *Pterocephalus hookeri* (C.B. Clarke) Höeck ethanolic extract presents analgesic and anti-inflammatory activities, and these activities were attributed to the fraction of the main constituents, the bis-iridoid type compounds [82].

From the analysis of some reviews involving iridoids activity, it can be noticed that a few examples, from which logonin **28** and swertiamarin **63** (Figure 3) can be highlighted, are being evaluated in vivo studies. Anti-inflammatory [83] and antidiabetic [84] evaluations of both the above-mentioned iridoids and the anti-advanced glycation end products formation potential of logon in **28** [85] are important examples, moreover if we consider the fact that these iridoids can be found in *Scabiosa* species.

Swertiamarin **63** is an interesting compound for which several in vivo studies were reported. The first example reports on its ability to reduce the sensitivity to painful stimuli [86], which is similar to the one showed by paracetamol. In the three in vivo studied models, swertiamarin **63** was shown to be active in a dose-dependent manner, but also shown to be safe up to 2000 mg/kg bw [86]. Later on, it was confirmed that swertiamarin 63 can be used to treat type II diabetes mellitus because it can regulate the peroxisome proliferator-activated receptor gamma (PPAR-γ) and increases insulin sensitivity [87]. Recently Mir and coworkers [88] demonstrated, in vivo, that swertiamarin **63** can inhibit both α-amylase and α-glucosidase which are enzymes involved in carbohydrate metabolism. This study accentuates the antidiabetic therapeutic potential of this iridoid glucoside.

In 2014, two interesting and complementary works of the Ignacimuthu research group [89,90], aiming to validated the medicinal properties of a plant used in Indian traditional medicine, evaluated the in vivo anti-inflammatory activity of swertiamarin **63**. The first aspect to be highlighted is the fact that no adverse effects were detected with a dose up to 500 mg/kg bw [89], however, the dosages used in the studies were much lower and also had beneficial effects. The combined assays (in vivo, in vitro and in silico) suggest that swertiamarin **63** anti-inflammatory effect is accomplished through the suppressing of pro-inflammatory mediators and inducing anti-inflammatory mediators such as helper T cells cytokines (Th2) [89]. Furthermore, the authors showed that swertiamarin **63** decreases the levels of nuclear factor kappa-light-chain-enhancer of activated B cells (NF-κB) and phospho-IκB alpha (p-IκBα), attenuates the release of both phospho-signal transducer and activator of transcription 3 (p-STAT3) and phospho-Janus kinase 2 (p-JAK2) levels [90]. Thus, swertiamarin **63** and/or its derivatives can become interesting therapeutics to treat rheumatoid arthritis.

Recently, this research group added more information about the swertiamarin 63 effects on and/or prevention of rheumatoid arthritis [91]. Again, the authors joined several methodologies to assess the biological activity, including an in vivo model (Freund’s complete adjuvant), which is the type of assessment that is discussed herein. Receptor activator of nuclear factor κB ligand (RANKL) and its receptor RANK, osteoprotegerin (OPG) and tartrate resistant acid phosphatase (TRAP) are recognized osteoclastogenis markers, a reason why their levels were measured in this study. The in vivo results showed that a treatment with swertiamarin **63** decreases the expression of the markers RANKL/RANK and TRAP and increases the OPG levels and these good results suggest that the anti-osteoclastogenic activity of swertiamarin **63** raises its potential use in rheumatoid arthritis treatment [91].

The antimicrobial activity of swertiamarin **63** was also reported [92]; however, the in vivo studies, as far as we could find, are limited. An interesting in vivo study was recently reported by Bodakhe and coworkers [93] where they disclose the synergistic effect of swertiamarin **63** against *Plasmodium berghei*. The results showed that the use of this iridoid improves the activity; however, its use to treat malaria should be investigated further.

Sweroside **62**, similar in structure (Figure 3 and Table 1) and in natural occurrence to swertiamarin **63** is, however, less evaluated in in vivo models. As far as we are aware, two in vivo studies were recently published, the evaluation of sweroside **62** ability to inhibit the body pigmentation and the tyrosinase activity, using zebrafish in vivo model [94], and inhibit human leukemia cell lines (HL-60) growth in xenograft mouse models [95]. Both studies are recent and preliminary, nevertheless are a confirmation that sweroside **62** biological properties may also be as remarkable as the ones found for swertiamarin **63**.

Naturally, our last example is logonin **28** (Figure 3 and Table 1), the other iridoid found in *Scabiosa* species that has been the focus of several in vivo studies. The first in vivo study that we could find involves the interesting antiamnesic activity [96] through the inhibition of acetylcholinesterase, result that indicates the potential therapeutic use of logonin **28** in Alzheimer’s disease treatment. This neuroprotective potential was observed by other research group [97] and later on was also detected in diabetic male rats [98]. More recently, was demonstrated the logonin **28** potential to be used in the treatment of neuromuscular diseases [99] through the increase of the survival motor neuron (SMN) protein level.

The logonin **28** beneficial effect on in vivo studies involving mice with induced diabetes was also observed in the diabetic nephropathy control [100,101]. Both works suggest that logonin **28** can be a good remedy to treat this disease, through the inhibition of connective tissue growth factor (CTGF) expression [100], or the inhibition of advanced glycation end-product (AGE) pathways [101]. In our opinion, the beneficial effects are evident but the medicinal implementation needs at least toxicological studies.

Our final examples are two, very recent works, that demonstrate the potential of logonin **28** to control inflammations. One article shows that this iridoid inhibits the substance P neurokinin-1 receptor and in doing so prevents the bladder hyperactivity [102]. Moreover, the mechanism of action seems to be through the downregulation of inflammatory leukocytes, decrease of induce intercellular adhesion molecule-1 (ICAM-1) expression and decrease of reactive oxygen species (ROS) production. All these aspects suggest an anti-inflammatory potential of logonin **28** [102]. The other example is a combination of in vitro and in vivo assays where the authors demonstrated that logonin **28** can relieve the inflammation stress [103].

The above mention findings for the chosen iridoids indicate that *Scabiosa* species medicinal use maybe due to these important bioactive secondary metabolites.

### 4.3. Pentacyclic Triterpenoid Type Metabolites

As can be seen in Figure 4, *Scabiosa* genus is rich in saponins where the main aglycones are oleanolic and pomolic acids, nonetheless, ursolic acid and hederagenin derivatives can also be found. These saponins in vivo assessments are scarce and the only aglycone until now isolated is the ursolic acid **65** (Figure 4), however, for the above mentioned aglycones, several in vivo studies reporting interesting results were published. For example, pomolic acid anti-inflammatory and apoptotic activities [104] and the antitumor activity of hederagenin [105] or macranthoside B, a natural hederagenin glycoside, [106] or hederagenin synthetic derivatives [107].

Nevertheless, ursolic and oleanolic acids are the most studied ones due to their recognized biological properties. If ursolic acid or its derivatives are not abundant in *Scabiosa* genus the same cannot be said about oleanolic acid and its glycoside derivatives, which are ubiquitous in this genus. Therefore, it is obvious that this genus can be an important source of this pentacyclic triterpenoid, reason why it is interesting to notice that recent biological assays involve oleanolic acid in vivo studies. In fact, the therapeutic potential of oleanolic acid was recently reviewed [108] and from that detailed work it is possible to conclude that indeed this natural compound is a good candidate to become a medicine. Due to this biological potential, the in vivo evaluations are increasing and in the last three years several publications involving the usual activities, such as antitumor [109,110,111], antidiabetic [112,113,114,115], anti-malarial [116] and anti-atherosclerosis [117] or the less common such as its beneficial effect on wound healing and regeneration [118] and the inhibition of matrix metalloproteinase-3 (MMP-3) production [119] have been published. It should be highlighted that this enzyme is involved in the articular cartilage destruction, thus oleanolic acid may be a potential drug to be used in the prevention of osteoarthritis cartilage damage [119].

Oleanolic acid has, however, a problem that might prevent its use in medicine; its low solubility in water and consequently its low bioavailability. Recent works have been devoted to solving this vital aspect [120,121,122,123] and some attention is being given to the use of nanoparticles [122,123]. Although, as was referred above, the genus *Scabiosa* is richer in this acid saponins, it cannot be ignored that the species can deliver oleanolic acid if used in the diet or be a source to isolate it.

## 5. Conclusions

At the end of this survey, it is possible to recognize the richness of *Scabiosa* genus in bioactive secondary metabolites. From which flavonoids and iridoids can be highlighted both from the biological properties previously revealed, but also for the in vivo assays already performed. In fact, from the secondary metabolites found in *Scabiosa* species these are the most evaluated ones. Moreover, these metabolites can validate some traditional uses but also can encourage other uses; in fact, these metabolites suggest that *Scabiosa* species can have interesting effects such as anti-inflammatory and anti-cancer activities, just to mention a few. Not only can these metabolites enlarge the traditional use of *Scabiosa* species but also can inspire the development of new drugs with therapeutic improvements.

Saponins are also abundant in the *Scabiosa* genus and are important secondary metabolites. However, their biological evaluations in vivo are restricted to their aglycones. This prompted us to suggest that these saponis should be evaluated to find out their biological potential and maybe find new drugs. These secondary metabolites can also be evaluated from the nutritional value and maybe prompt the use of *Scabiosa* species in food preparations.

It is also important to highlight the fact that, in the last few years, several new natural compounds were isolated from the *Scabiosa* species. Furthermore, the survey herein presented also demonstrates that several species are, from the phytochemical point of view, neglected. These findings should encourage further studies that can reveal the medicinal potential of this genus species. Indeed, *Scabiosa* species may be a good source of new bioactive natural compounds.

## Figures and Tables

**Table 1:** Secondary metabolites isolated from *Scabiosa* species.

Nº	Name ^1^	Plant Part (Solvent)	Species
***Flavonoid Derivatives***			
1	Apigenin ^a^	Whole plant (MeOH) [28] Whole plant (EtOH) [29]	*S. tenuis* [28] *S. stellata* [29]
2	Astragallin ^b^	Flowering plants (CH_2_Cl_2_/MeOH) [23]	*S. hymettia* [23]
3	Cynaroside ^b^	Whole plant (MeOH or ButOH) [28] Aerial (leaves and stems) parts (EtOH) [22] Epigeal part (MeOH) [30]	*S. atropurpurea* [22] *S. olgae* [30] *S. tenuis* [28] *S. argentea* [28]
4	Diosmetin-7-*O*-β-glucoside ^b^	Whole plant (ButOH) [28]	*S. argentea* [28]
5	Hyperin ^3,b^	Whole plant (EtOH) [2]	*S. stellata* [2]
6	Isoorientin ^b^	Whole plant (EtOH) [2,29] Whole plant (ButOH) [28]	*S. argentea* [28] *S. stellata* [2,29]
7	Isovitexin ^b^	Whole plant (MeOH) [28]	*S. tenuis* [28]
8	Kaempferol-3-*O*-[3-*O*-acetyl-6-*O*-(*E*)-*p*-coumaroyl]-β-d-glucoside ^b^	Flowering plants (CH_2_Cl_2_/MeOH) [23] Whole plant (EtOH) [31]	*S. hymettia* [23] *S. stellata* [31]
9	Lucenin ^2,b^	Whole plant (EtOH) [29]	*S. stellata* [29]
10	Luteolin ^a^	Aerial (leaves and stems) parts (EtOH) [22] Whole plant (EtOH) [29] Whole plant (MeOH) [28]	*S. atropurpurea* [22] *S. tenuis* [28] *S. stellata* [29]
11	Luteolin-7-*O*-β-gentiobioside ^c^	Whole plant (MeOH or ButOH) [28]	*S. argentea* [28] *S. tenuis* [28]
12	Luteolin-7-*O*-rutinoside ^c^	Aerial (leaves and stems) parts (EtOH) [22]	*S. atropurpurea* [22]
13	Quercetin ^a^	Whole plant (ButOH) [28]	*S. argentea* [28]
14	Quercetin-3-*O*-arabinoside ^b^	Whole plant (ButOH) [28]	*S. argentea* [28]
15	Quercetin-3-*O*-galactoside ^b^	Whole plant (ButOH) [28]	*S. argentea* [28]
16	Swertiajaponin ^b^	Whole plant (EtOH) [2]	*S. stellata* [2]
17	Tamarixetin 3-β-l-rhamnosyl-(1→2)[β-l-rhamnosyl-(1→6)]β-d-glucoside] ^d^	Whole plant (EtOH) [29]	*S. stellata* [29]
18	Tiliroside ^b^	Whole plant (EtOH) [29]	*S. stellata* [29]
19	Vitexin ^b^	Whole plant (MeOH) [28]	*S. tenuis* [28]
*Terpenoid derivatives*			
20	7-*O*-(*E*-Caffeoyl)sylvestroside I ^c^	Whole plant (EtOH) [2]	*S. stellata* [2]
21	7-*O*-(*E*-*p*-Coumaroyl)sylvestroside I ^c^	Whole plant (EtOH) [2]	*S. stellata* [2]
22	Cantleyoside ^c^	Flowers (MeOH) [32] Whole plant (MeOH) [33]	*S. atropurpurea* [32] *S. variifolia* [33]
23	Eustomoruside ^b^	Whole plant (EtOH) [2]	*S. stellata* [2]
24	Eustomoside ^b^	Whole plant (EtOH) [2]	*S. stellata* [2]
25,26	Hookeroside A ^g^ and B ^h^	Whole plant (MeOH) [34]	*S. tschilliensis* [34]
27	Loganic acid ^b^	Flowering plants (CH_2_Cl_2_/MeOH) [23] Flowers (MeOH) [32] Whole plant (MeOH) [33]	*S. hymettia* [23] *S. atropurpurea* [32] *S. variifolia* [33]
28	Loganin ^b^	Flowering plants (CH_2_Cl_2_/MeOH) [23] Flowers (MeOH) [32] Whole plant (MeOH) [33]	*S. hymettia* [23] *S. atropurpurea* [32] *S. variifolia* [33]
29	Palustroside III ^d^	Whole plant (EtOH) [31]	*S. stellata* [31]
30 to 40	Scabiosaponin A ^g^, B ^h^, C ^h^, D ^f^, E ^f^, F ^f^, G ^g^, H ^g^, I ^f^, J ^f^ and K ^g^	Whole plant (MeOH) [34]	*S. tschilliensis* [34]
41 to 48	Scabiostellatosides A ^g^, B ^g^, C ^h^, D ^h^, E ^h^, F ^h^, G ^e^ and H ^d^	Whole plant (EtOH) [31]	*S. stellata* [31]
49 to 52	Scabrioside A ^d^, B ^e^, C ^e^, and D ^f^	Roots (MeOH) [35]	*S. rotata* [35]
53	Septemfidoside ^c^	Whole plant (EtOH) [2]	*S. stellata* [2]
54 to 60	Songoroside A ^b^, C ^c^, E ^d^, G ^e^, I ^f^, M ^g^ and O ^h^	Roots (EtOH) [36]	*S. songarica*^2^ [36]
61	Stigmasterol ^a^	Whole plant (hexane) [37]	*S. stellata* [37]
62	Sweroside ^b^	Whole plant (EtOH) [2] Flowers (MeOH) [32] Whole plant (MeOH) [33]	*S. atropurpurea* [32] *S. variifolia* [33] *S. stellata* [2]
63	Swertiamarin ^b^	Flowering plants (CH_2_Cl_2_/MeOH) [23] Flowers (MeOH) [32] Whole plant (MeOH) [33]	*S. hymettia* [23] *S. atropurpurea* [32] *S. variifolia* [33]
64	Sylvestroside I ^c^	Whole plant (EtOH) [2]	*S. stellata* [2]
65	Ursolic acid ^a^	Whole plant (EtOH) [31] Whole plant (hexane) [37]	*S. stellata* [32,37]
66	β-Sitosterol-β-d-glucoside ^b^	Whole plant (hexane) [37]	*S. stellata* [37]

^1^ Compounds are presented in alphabetic order; ^2^ Although the authors indicate that they study the species *Scabiosa soongorica* Schrenk, we think that the current name is *Scabiosa songarica* Schrenk; ^3^ This name is a synonym of hyperoside, herein is indicated the name adopted by the authors [2]; ^a^ isolated as aglycones; ^b^ isolated as monoglycosides; ^c^ isolated as diglycosides; ^d^ isolated as triglycosides; ^e^ isolated as tetraglycosides; ^f^ isolated as pentaglycosides; ^g^ isolated as hexaglycosides; ^h^ isolated as heptaglycosides.
